# Protective effect of the novel calcineurin inhibitor voclosporin in experimental colitis

**DOI:** 10.3389/fmed.2023.1177450

**Published:** 2023-06-09

**Authors:** Aylin Lindemann, Dominik Roth, Kristina Koop, Clemens Neufert, Sebastian Zundler, Raja Atreya, Markus F. Neurath, Moritz Leppkes

**Affiliations:** ^1^Department of Medicine 1, Universitätsklinikum Erlangen, Friedrich-Alexander-University Erlangen-Nürnberg (FAU), Erlangen, Germany; ^2^Deutsches Zentrum Immuntherapie (DZI), Erlangen, Germany

**Keywords:** IBD, calcineurin inhibitors, ulcerative colitis, steroid refractory colitis, voclosporin

## Abstract

**Background and aims:**

Acute severe steroid-refractory ulcerative colitis remains a medically challenging condition with frequent need of surgery. It can be treated with the calcineurin inhibitor cyclosporine A with the need for therapeutic drug monitoring and significant toxicity. Recently, a novel calcineurin inhibitor, voclosporin, has been approved for the treatment of lupus nephritis with no need for therapeutic drug monitoring and an improved long-term safety profile. However, the therapeutic effect of voclosporin in acute severe steroid-refractory ulcerative colitis is still uncertain. We aimed to assess the therapeutic potential of voclosporin to ameliorate inflammation in an experimental model of colitis.

**Methods:**

We used the dextran sodium sulfate-induced model of colitis in C57BL/6 J wildtype mice treated with either cyclosporine A, voclosporin or solvent control. We employed endoscopy, histochemistry, immunofluorescence, bead-based multiplex immunoassays and flow cytometry to study the therapeutic effect of calcineurin inhibitors in a preventive setting.

**Results:**

Acute colitis was induced by dextran sodium sulfate characterized by weight loss, diarrhea, mucosal erosions and rectal bleeding. Both cyclosporine A and voclosporin strongly ameliorated the course of disease and reduced colitis severity in a similar manner.

**Conclusion:**

Voclosporin was identified as biologically effective in a preclinical model of colitis and may be a potential therapeutic option in treating acute severe steroid-refractory ulcerative colitis.

## Introduction

Acute severe ulcerative colitis remains a challenging clinical situation with unmet clinical need. In the setting of steroid-refractory disease up to 40% of patients require colectomy as a measure of last resort (1). Cyclosporine A has been used for some decades and may achieve induction of remission in steroid-refractory UC in up to 80% of patients (2). Additionally, the anti-TNF antibody infliximab is a successfully used therapeutic option in this setting (3). The CYSIF and CONSTRUCT trials compared the efficacy of the anti-TNF agent infliximab and cyclosporine A in head-to-head trials and observed that both represent similarly effective options (4, 5). However, both treatment options leave a significant proportion of up to 40% of patients who fail medical therapy. Both treatment options have similar outcomes on a long-term perspective (6). Thus, even after more than 30 years of use, calcineurin inhibitors have not been surpassed in treatment efficacy in the setting of acute steroid-refractory ulcerative colitis. On the other hand, use of cyclosporine A is hampered by significant toxicities including nephrotoxicity, hypertension, dyslipidemia and hyperuricemia (7).

A novel calcineurin inhibitor, voclosporin (ISA247) has been approved by the FDA for the treatment of lupus nephritis in January 2021 and the EMA in September 2022 based in part on the results of the AURORA 1 trial (8). Differences in its pharmacokinetic potency, enhanced binding to calcineurin and reduced load of potentially toxic metabolites may lead to reduced cardiac and renal toxicities of voclosporin as compared to cyclosporine A (9). Additionally, in contrast to cyclosporine A, there is an improved pharmacokinetic profile potentially omitting the need of therapeutic drug monitoring of voclosporin (10). We hypothesized a role for this novel calcineurin inhibitor for the treatment of acute steroid-refractory ulcerative colitis and performed pilot preclinical studies employing an experimental model of colitis.

## Materials and methods

### Mice

We used female C57BL/6 J mice for DSS-induced colitis experiments. Mice aged 12–16 weeks were used for experimental procedures. All mice were kept under specific pathogen-free conditions at the animal facility of the University of Erlangen. Experimental procedures were approved by the local committees of Lower Franconia (AZ 55.22532-2-358).

### Experimental models of disease

Acute colitis was induced by the administration of 3% dextran sodium sulfate (DSS, 36–50 kD; MP Biotech, Santa Ana, CA, United States) to the drinking water of mice (*N* = 10 mice per group). Weight and clinical features were documented throughout the experiment. Daily i. p. injections of solvent (3% ethanol in sunflower oil) were applied 3 days before start of DSS administration to enable adaptation to treatment. From the beginning of DSS administration until the end of the experiment, mice were injected daily with 10 mg/kg cyclosporine A, voclosporin or solvent control. To avoid cage-dependent effects, treatment was administered in mixed cages. Mice were assigned to the different groups ensuring similar body weight mean for each group on day 0. Endoscopic analysis of the colonic mucosa was performed on day 8 after start of DSS administration. Rectal bleeding and stool consistency were monitored daily. Mice were euthanized if a weight loss of 20% occurred during the course of the disease or sacrificed at day 9 (*N* = 5 mice per group) or day 14 (*N* = 5 mice per group) for further examinations. COLOVIEW high-resolution mouse video endoscopic system (Karl Storz, Tuttlingen, Germany) was used for mouse colonoscopy. Mice were sacrificed at the indicated time points and colon tissue was dissected. Blood samples were collected and blood counts performed. Tissue samples were subjected to further analyses using RNA isolation techniques as well as analyses by histochemistry.

### Endoscopic scoring

Scoring of the inflamed colonic mucosa was performed during rectoscopy at day 8 of DSS treatment. Translucency, granularity, fibrin, ulceration, vascularity, diarrhea and mucosal bleeding were assessed for each animal.

### Cell isolation procedures

Human peripheral blood mononuclear cells (PBMCs) from healthy donors were isolated from whole blood anticoagulated by EDTA using Ficoll technique after ethical approval of local authorities (approval numbers: 40_16B, 426_20B). Isolated cells were washed with sterile PBS buffer and subjected to magnetic activated cell sorting (MACS, Miltenyi Biotec) for enrichment of CD4^+^ T cells.

### Stimulation of T cells

Enriched CD4^+^ T cells in an IMDM-based medium supplemented with 10% FCS and 1% penicillin/streptomycin were seeded in culture plates and stimulated with α-CD3 (1 μg/mL) and α-CD28 (2 μg/mL) antibodies or phorbol-12-myristat-13-acetat (PMA, 25 ng/mL) and ionomycin (1 μg/mL). Cells were subsequently incubated in presence or absence of either cyclosporine A (8.3 μM) or voclosporin (8.3 μM) for 48 h. Subsequently, supernatants were collected for cytokine analyses and cell pellets were collected in RNA lysis buffer for further processing.

### Flow cytometric analyses

MACS-enriched human CD4^+^ T cells stimulated with α-CD3 (1 μg/mL) and α-CD28 (2 μg/mL) antibodies or phorbol-12-myristat-13-acetat (PMA, 25 ng/mL) and ionomycin (1 μg/mL) and cultured for 48 h in the presence or absence of cyclosporine A, voclosporin or solvent control were subjected to staining with fluorophore-coupled antibodies (live/dead staining using Zombie Aqua dye, anti-CD4, anti-CD25, anti-CD44, and anti-CD69). Subsequent flow cytometric analyses were performed using a LSRFortessa™ Cell Analyzer and FlowJo software (BD Biosciences, Franklin Lakes, NJ, United States).

### Measurement of cytokine concentrations

Supernatants of stimulated T cells were collected after 48 h of stimulation, centrifuged and analyzed for concentration of cytokines. Analysis was performed using the LEGENDplex™ HU Th Cytokine Panel (12-plex; BioLegend, San Diego, CA, United States) and measured using an Accuri™ C6 Flow Cytometer (BD Biosciences, Franklin Lakes, NJ, United States).

### Real-time quantitative PCR

Tissue or T cell RNA was isolated after directly freezing samples in liquid nitrogen in lysis buffer of the peqGOLD Total RNA Kit (Peqlab, Erlangen, Germany). RNA quantification was performed using Nanodrop technology (Thermo Scientific, Wilmington, DE, United States). Reverse transcription into cDNA was performed using the BioRad iScript cDNA synthesis Kit (Bio-Rad Laboratories, Munich, Germany). As a quality control, RT-PCR for *Actb* or *ACTB* was performed and only samples with a positive PCR product after 25 cycles were used for subsequent qPCR studies. qPCR was performed using human QuantiTect Primer Assays for *HPRT, CASP8, IFNG*, *IL2, IL10*, *IL17a*, *IL17f*, *ITK*, *MALT1*, and *TNF* (Qiagen, Hilden, Germany). Murine gene expression was analyzed with QuantiTect Primer Assays for *Hprt, Il1b*, *Ptgs2*, and *S100a9* (Qiagen, Hilden, Germany). Analyses were performed using SensiFAST™ SYBR® No-ROX Kit (Meridian Bioscience) on the Bio-Rad CFX Connect system (Bio-Rad Laboratories, Munich, Germany). Expression was calculated relative to the housekeeping gene *Hprt*/*HPRT* using the delta threshold cycle (ΔCt) algorithm. Fold difference to control treated animals or unstimulated control, respectively, was calculated as a ratio to the respective control mean.

### Immunofluorescence techniques

Histological staining was performed on paraffin-embedded sections with the classical hematoxylin–eosin (H&E) staining procedure. Immunofluorescence on paraffin-embedded slides was performed using overnight incubation with primary Abs specific for E-Cadherin (BD, Germany, 1:200, clone 36/E-Cadherin) and neutrophil elastase (NE; Abcam, Cambridge, United Kingdom, 1:200, polyclonal). Detection was performed using either biotinylated secondary Abs (goat anti-rabbit, Dianova, 1:500, polyclonal) and TSA Fluorescein/Cy3 kits (PerkinElmer, Waltham, MA, United States) or Dylight-550-labeled streptavidin (1:200). Glycoprotein staining in goblet cells was performed using fluorescein-labeled *Ulex Europaeus* Agglutinin I (UEA I, Vector Laboratories, 1:100). Before examination, the nuclei were counterstained with Hoechst 33342 (Invitrogen Molecular Probes, Karlsruhe, Germany). Images were recorded on Leica fluorescence microscope (Leica, Germany).

### Calculation of ulcerated area and neutrophil influx

Tissue sections stained with H&E, UEA I or Abs specific for E-Cadherin or NE were used for a blinded morphometric analysis calculating the area affected by ulceration relative to the total sectional mucosal surface or the number of NE-positive cells per section.

### Statistical analysis

Data were analyzed as indicated in the figure legends using analysis of variance (ANOVA) with parametric and non-parametric tests and post-hoc correction for multiple comparisons assisted by GraphPad Prism.

### Data availability

Original data acquired during the course of the study is made available for third-party analysis upon request to the corresponding author.

## Results

### Calcineurin inhibition by either cyclosporine A or voclosporin abrogates the production of multiple T cell-derived cytokines

**Figure 1:**
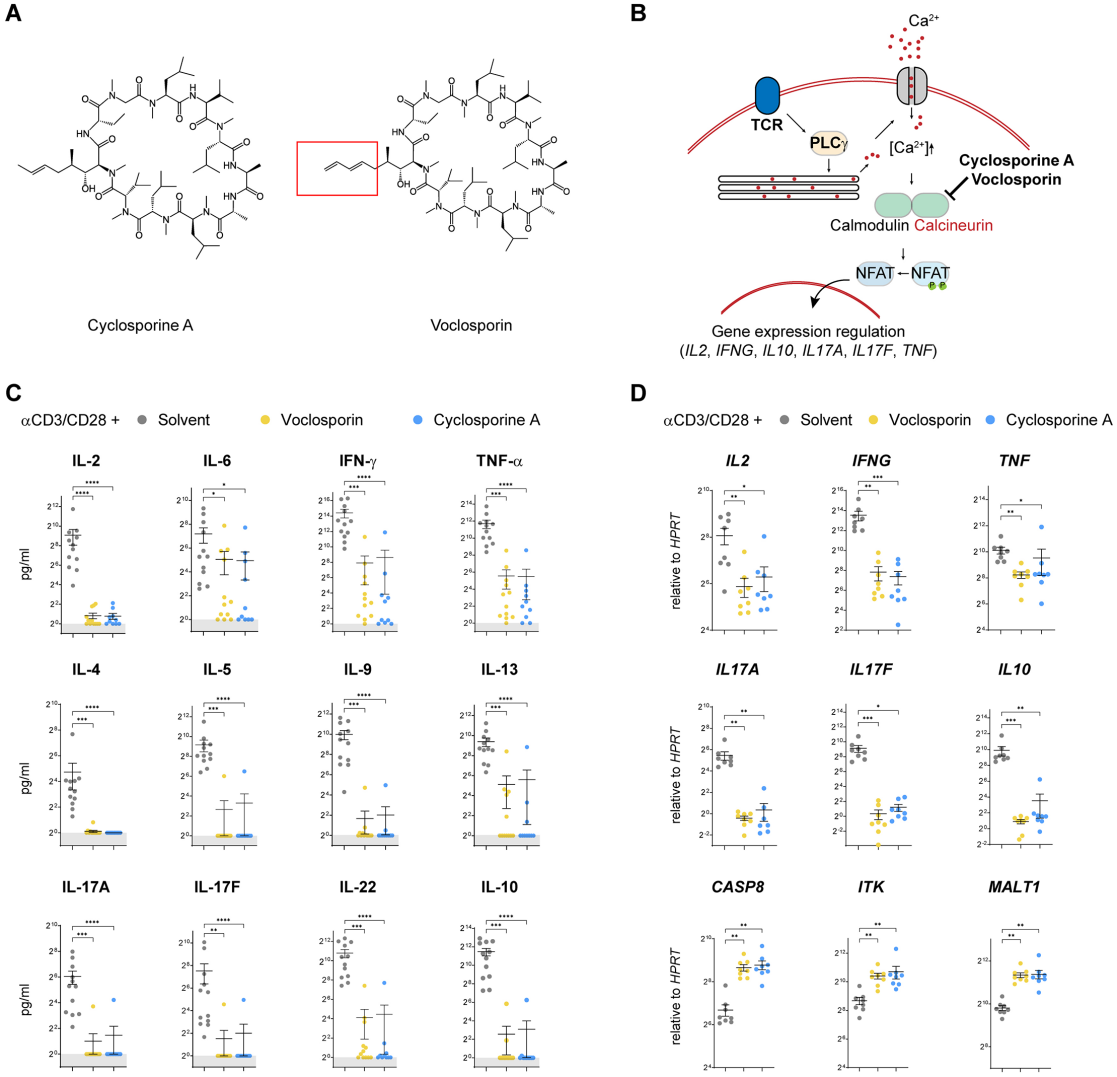
Calcineurin inhibition by either cyclosporine A or voclosporin abrogates the production of multiple T cell-derived cytokines. **(A)** The molecular structure of the calcineurin inhibitors cyclosporine A and voclosporin is presented and its structural difference at the amino acid 1—side chain position is highlighted. **(B)** A working model of the proposed mechanism of action of the calcineurin inhibitors is presented. **(C,D)** Human peripheral blood T lymphocytes were isolated from normal healthy donors and stimulated with anti-CD3/CD28 for 48 h in the presence or absence (solvent control, gray) of the calcineurin inhibitors voclosporin (10 μg/mL, yellow) or cyclosporine A (10 μg/mL, blue; *N* = 10 donors, pooled analysis of at least three independent experiments). Consecutively, culture supernatants were analyzed for cytokine secretion and T cell RNA was isolated and subjected to qPCR analyses. **(C)** In stimulated T cells, ample amounts of IL-2, IL-6, IFN-γ, TNF-α, IL-4, IL-5, IL-9, IL-13, IL-17A, IL-17F, IL-22, and IL-10 are produced which is abrogated in the presence of either voclosporin or cyclosporine A. **(D)** qPCR analysis of *IL2*, *IFNG*, *TNF*, *IL17A*, *IL17F*, and *IL10* revealed that not only secretion but also induction of transcription of these cytokines is abrogated in the presence of either voclosporin or cyclosporine A. In contrast, expression of *CASP8*, *ITK* and *MALT1* is elevated in the presence of either calcineurin inhibitor (*N* = 8, pooled analysis of at least three independent experiments; ****p < 0.0001, ***p < 0.001, **p < 0.01, *p < 0.05, Kruskal-Wallis-Test with Dunn’s correction).

Cyclosporine A and voclosporin are both cyclic peptides including 11 amino acids (Figure 1A). Voclosporin is modified in the amino acid 1 residue as compared to its ancestral drug. Cyclosporine A and voclosporin are known to inhibit calcineurin-mediated dephosphorylation of the transcription factors NFAT1-4 downstream of T cell receptor stimulation, which prevents nuclear translocation of these transcription factors and thus affects gene expression regulation of multiple cytokines, e.g., IL-2, IFN-γ and others (Figure 1B). In order to test the efficacy of voclosporin we assessed the effect of both voclosporin and cyclosporine A in human CD4^+^ T lymphocytes isolated from peripheral blood as a model cell type which is affected by calcineurin inhibition. T lymphocytes were polyclonally stimulated either by anti-CD3/CD28 antibodies (Figures 1C,D) or by phorbol myristate acetate and ionomycin (Supplementary Figure S1). Both cytokine concentrations in culture supernatants and gene transcription were analyzed. These analyses showed a markedly reduced secretion of all cytokines measured in the presence of either voclosporin or cyclosporine A, including IL-2, IL-6, IFN-γ, TNF-α, IL-4, IL-5, IL-9, IL-13, IL-17A, IL-17F, IL-22, and IL-10 (Figure 1C). Additionally, the transcription of *IL2*, *IFNG*, *TNF*, *IL17A*, *IL17F*, and *IL10* was significantly blocked by either voclosporin or cyclosporine A (Figure 1D). In contrast, *CASP8*, *ITK*, and *MALT1* expression was increased in the presence of either calcineurin inhibitor as compared to solvent control-treated cells (Figure 1D). These effects occurred irrespective of the stimulatory agents after either anti-CD3/CD28 (Figures 1C,D) or PMA/ionomycin stimulation (Supplementary Figures S1A,B). Thus, both calcineurin inhibitors strongly blocked cytokine expression and production by T lymphocytes *in vitro*.

### Calcineurin inhibition by either cyclosporine A or voclosporin modulates expression of surface activation markers upon TCR stimulation

**Figure 2:**
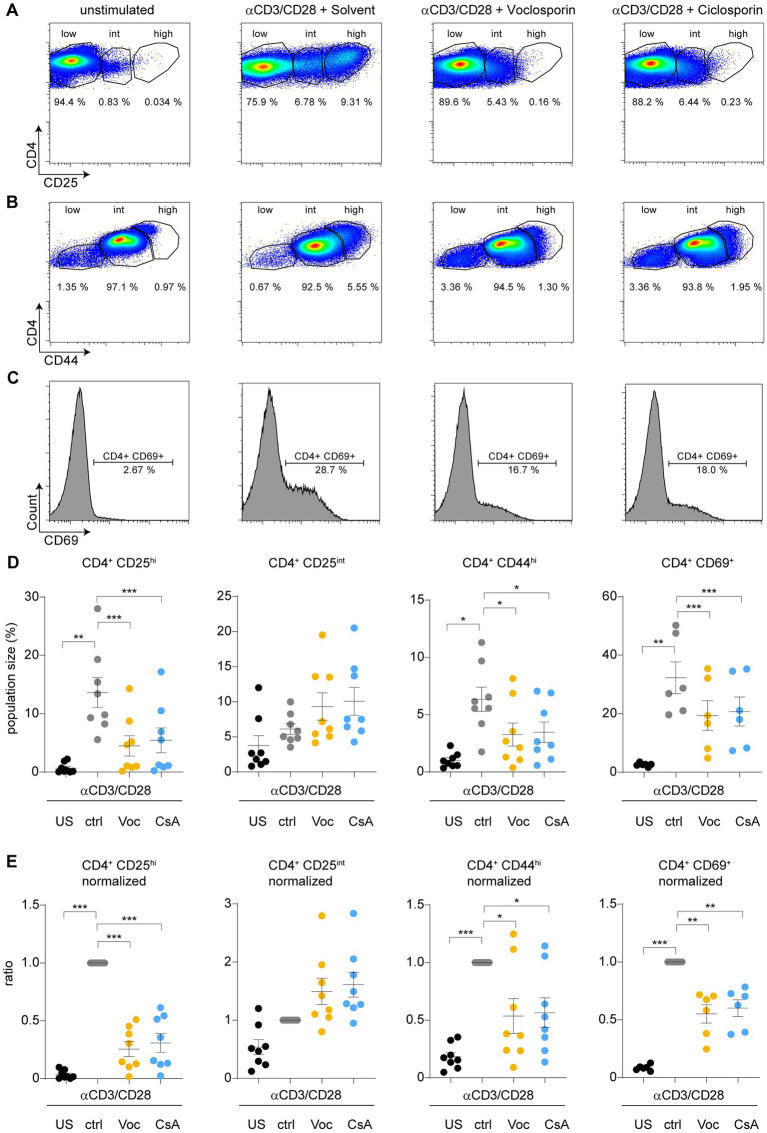
Calcineurin inhibition by either cyclosporine A or voclosporin modulates expression of surface activation markers upon TCR stimulation. CD4^+^ T lymphocytes were cultured without (unstimulated, US) or with anti-CD3/28 for 48 h in the absence (solvent control, ctrl, gray) or presence of either voclosporin (Voc, 10 μg/mL, yellow) or cyclosporine A (CsA, 10 μg/mL, blue). Consecutively, surface activation marker expression was assessed by flow cytometry. **(A)** Representative images of flow plots of CD4^+^ lymphocytes left unstimulated or stimulated with anti-CD3/CD28 in the presence of the calcineurin inhibitors or solvent control are depicted demonstrating that **(A)** CD25, **(B)** CD44, and **(C)** CD69 are strongly upregulated upon TCR stimulation, which is reduced in the presence of either calcineurin inhibitor. **(D)** Quantitative analysis of CD4^+^CD25^hi^, CD4^+^CD25^int^, CD4^+^CD44^hi^, and CD4^+^CD69^+^ cell populations of a pooled analysis of *N* = 8 healthy donors derived from at least three independent experiments are presented **(E)** Results of analyses as in **(D)** are presented after normalization to stimulated cells with solvent control. Please appreciate the consistently inhibited surface expression of CD25, CD44, and CD69 (US, unstimulated; ctrl, control; Voc, Voclosporin; CsA, Cyclosporine A; ***p < 0.001, **p < 0.01, *p < 0.05, ANOVA with Dunnett’s correction).

In a next step we evaluated surface activation marker expression of T lymphocytes stimulated in the presence or absence of either voclosporin or cyclosporine A. Polyclonal stimulation with anti-CD3/CD28 induced the marked upregulation of CD25 on CD4^+^ T lymphocytes. In the presence of either voclosporin or cyclosporine A this strong upregulation was attenuated. T cells still responded to anti-CD3/CD28 as evidenced by increased CD4^+^CD25^int^ cells, but CD4^+^CD25^hi^ cells were largely absent (Figures 2A,D,E). In accordance to the increase in CD25 expression, both CD44 (Figure 2B) and CD69 (Figure 2C) surface expression increased in response to polyclonal stimulation. Both calcineurin inhibitors significantly blocked surface expression of CD44 (Figures 2B,D,E) and CD69 (Figures 2C–E). Quantitative analyses of T cell surface marker expression are presented as absolute frequencies (Figure 2D) and normalized to control-stimulated cultures (Figure 2E). Thus, both calcineurin inhibitors consistently blocked T cell activation as evidenced by surface expression of classical activation markers.

### Calcineurin inhibition strongly ameliorates the clinical course of acute severe colitis

**Figure 3:**
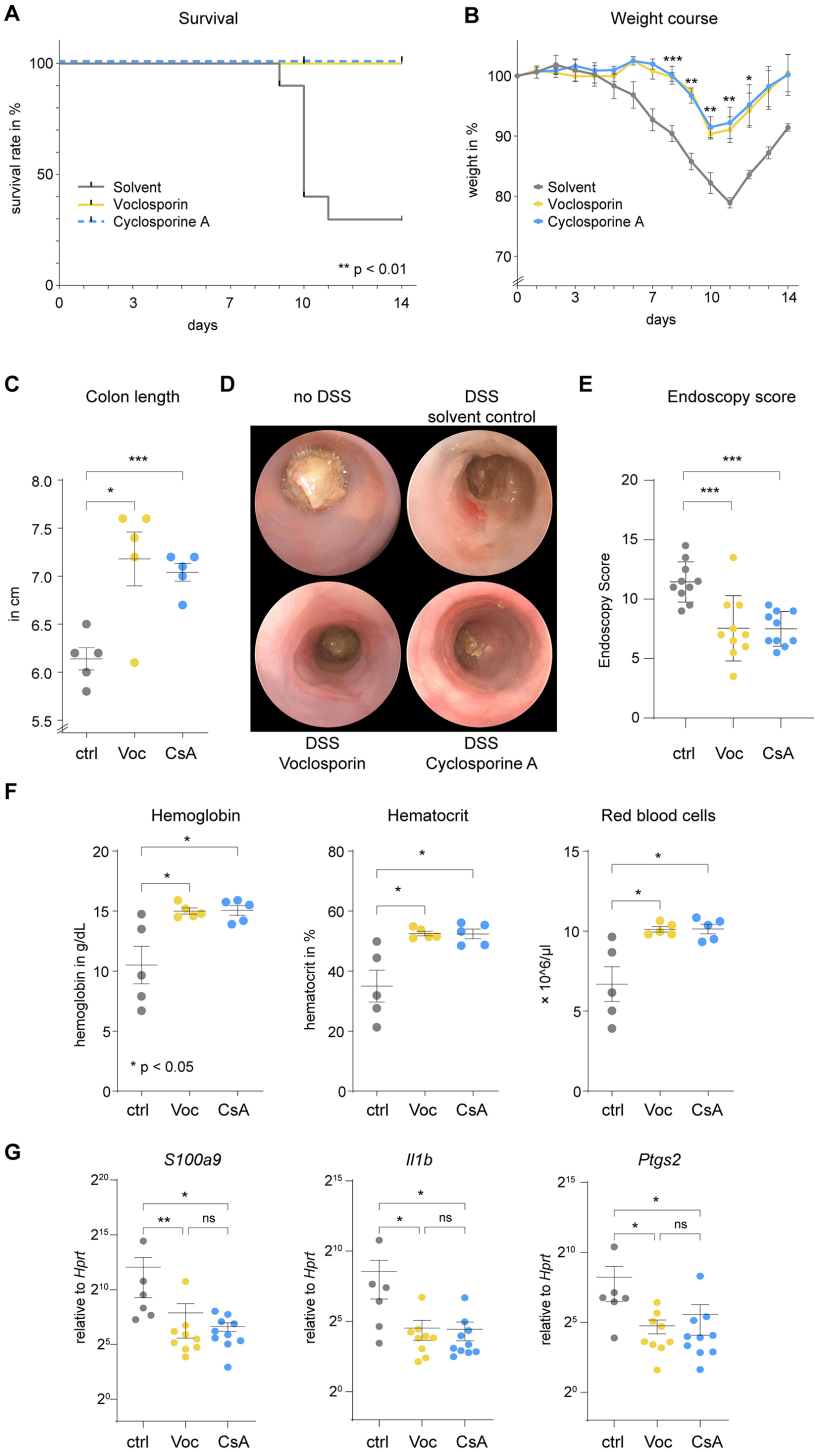
Calcineurin inhibition strongly ameliorates the clinical course of acute severe colitis. C57BL/6 J mice were subjected to acute DSS-induced colitis. **(A)** Lethality of mice (*N* = 10 mice per group), interpreted by the number of mice suffering a weight loss of >20%, was abrogated by either voclosporin (Voc, yellow) or cyclosporine A (CsA, blue) as compared to solvent control [ctrl, gray; **p < 0.01, Log-rank test (Mantel-Cox), accounted for multiple comparisons]. **(B)** The relative weight over time is depicted. Please appreciate the reduction in weight loss induced by calcineurin inhibition (***p < 0.001, **p < 0.01, *p < 0.05, ANOVA with Dunnett’s correction). **(C)** Mice sacrificed on day 9 (*N* = 5 mice per group) showed shortening of the colon in the course of DSS-induced colitis. Colon length was significantly longer when mice were treated with either voclosporin or cyclosporine A (***p < 0.001, *p < 0.05, Brown-Forsythe and Welch Test). **(D)** Mini-endoscopy was performed on day 8 of the experiment (*N* = 10 mice per group). In DSS-treated mice diarrhea occurs and mucosal erosions can be appreciated in the murine colon. Diarrhea was reduced and less erosions were visible upon endoscopy in voclosporin- or cyclosporine A-treated mice as compared to solvent control-treated mice. **(E)** Grading of colitis severity by endoscopy was performed and revealed less colitis severity in voclosporin- and cyclosporine A-treated mice as compared to solvent control-treated mice (*N* = 10 mice per group; ***p < 0.001, ANOVA with Dunnett’s correction). **(F)** Complete blood counts of mice were performed on day 9. Hemoglobin, hematocrit and red blood cell count were consistently reduced in solvent control treated mice as compared to voclosporin- or cyclosporine A-treated mice (*N* = 5 mice per group; *p < 0.05, Kruskal-Wallis Test with Dunn’s correction). **(G)** RNA was isolated from inflamed colon tissues of DSS-treated mice and qPCR was performed demonstrating significantly reduced levels of *S100A9*, *Il1b* and *Ptgs2* in voclosporin- or cyclosporine A-treated colon tissues (*N* = 6–10 mice per group; ctrl, control; Voc, Voclosporin; CsA, Cyclosporine A; *p < 0.05, Kruskal-Wallis Test with Dunn’s correction).

Next, we assessed the effect of voclosporin and cyclosporine A in experimental colitis. We chose the dextran sodium sulfate-model of acute colitis, which shares many clinical features with acute severe ulcerative colitis including diarrhea, rectal bleeding and weight loss. DSS treatment of mice induced marked colitis characterized by diarrhea, rectal bleeding and weight loss in solvent control-treated mice. Treatment with either voclosporin or cyclosporine A strongly improved survival (Figure 3A) and reduced weight loss (Figure 3B). Shortening of the colon is associated with increased disease severity. Treatment with either voclosporin or cyclosporine A strongly reduced bowel shortening during colitis (Figure 3C). Mini-endoscopy of mice revealed marked mucosal erosions and diarrhea in DSS-treated mice subjected to solvent control. Treatment with either voclosporin or cyclosporine A diminished colitis severity as evidenced by endoscopy (Figures 3D,E; Supplementary Video 1). Reduced mucosal blood loss and colitis severity was also evident in complete blood counts of mice: both voclosporin and cyclosporine A-treated mice had normal levels of hemoglobin, hematocrit and red blood cells, whereas solvent control-treated mice suffered blood loss in the course of DSS-induced colitis (Figure 3F). Reduced colitis severity was also reflected in differential tissue mRNA expression: mucosal transcript levels of *S100a9*, *Il1b* and *Ptgs2* were significantly reduced in DSS-induced colitis when calcineurin inhibitors were applied (Figure 3G).

### Calcineurin inhibition protects from mucosal erosions in acute severe colitis

**Figure 4:**
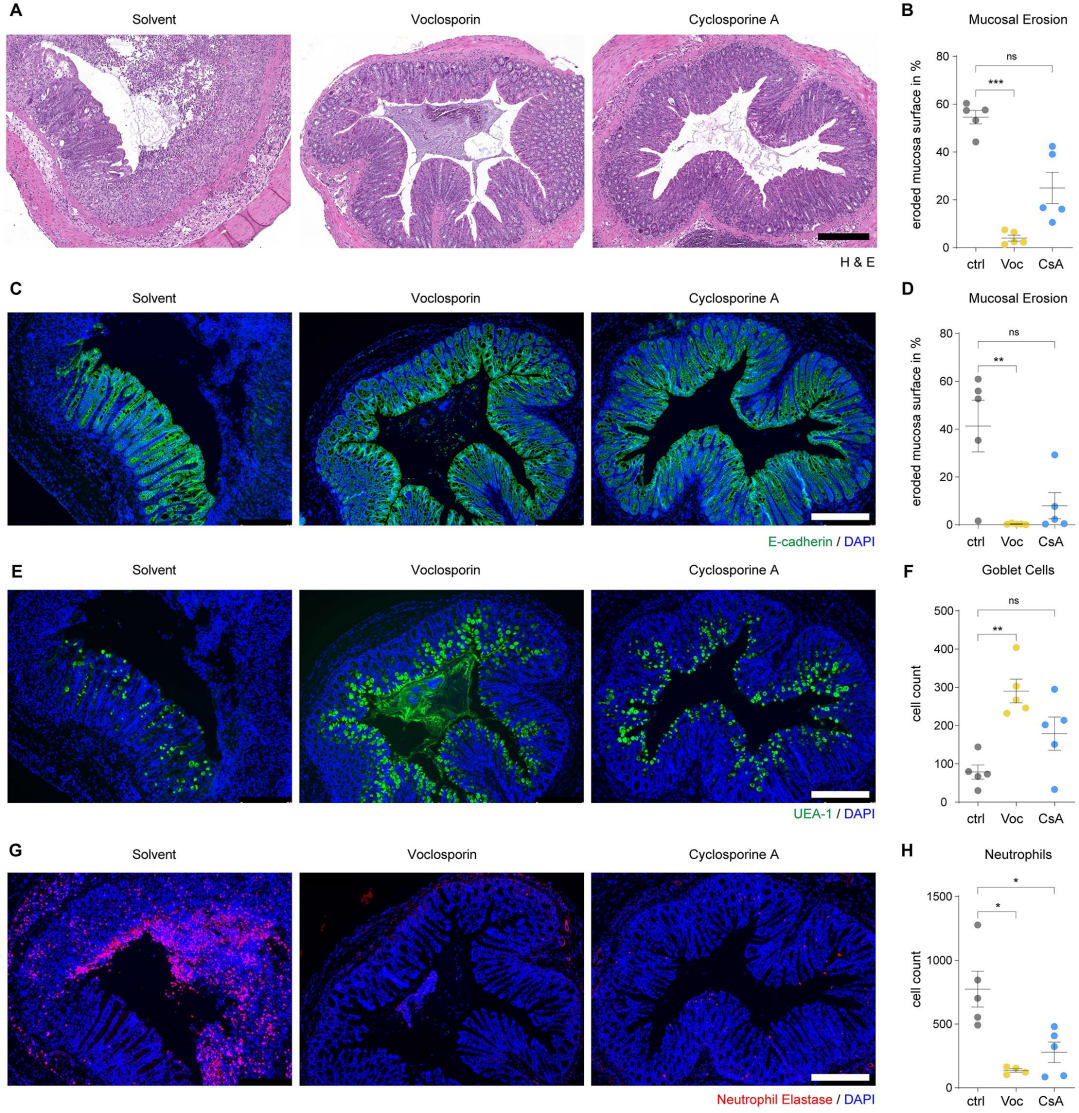
Calcineurin inhibition protects from mucosal erosions in acute severe colitis. Paraffin-embedded colon tissue samples of mice subjected to DSS-induced colitis and treated with either voclosporin (Voc, yellow), cyclosporine A (CsA, blue) or solvent control (ctrl, gray) were processed for histology and immunofluorescence analyses. **(A)** H&E staining shows the frequent presence of mucosal erosions in DSS-treated mice which is strongly reduced by treatment with either voclosporin or cyclosporine A. Representative images are shown. **(B)** The eroded mucosal surface as quantified on tissue sections as in **(A)** was significantly reduced by treatment with either voclosporin or cyclosporine A (*N* = 5 mice per group). **(C)** Immunofluorescence of E-Cadherin was performed to assess the epithelial layer of DSS-treated mice. While solvent control-treated mice showed marked disruptions of the epithelial layer, the epithelial layer in voclosporin- or cyclosporine A-treated mice was largely intact. Representative images are shown. **(D)** The quantification of the eroded mucosal surface as characterized in **(C)** underlines the significantly reduced occurrence of mucosal erosions in the presence of either voclosporin or cyclosporine A (*N* = 5 mice per group). **(E)** Goblet cell content of the intestinal epithelium was studied by means of UEA-1 immunofluorescence. Representative images are shown. **(F)** Goblet cells are lost in the course of acute DSS-induced colitis but calcineurin inhibition rescues goblet cell loss. Quantification of goblet cells per tissue sections as in **(E)** was performed (*N* = 5 mice per group). **(G)** Infiltration of neutrophil granulocytes was assessed by immunofluorescent detection of neutrophil elastase (NE). A marked infiltration of neutrophil granulocytes to the bowel wall and the colon lumen was observed in DSS-treated mice. Application of either voclosporin or cyclosporine A strongly reduced the infiltration of neutrophils associated with mucosal damage. **(H)** Quantification of neutrophils based on **(G)** reveals the significantly reduced infiltration of neutrophils in the presence of either voclosporin or cyclosporine A (*N* = 5 mice per group; ctrl, control; Voc, Voclosporin; CsA, Cyclosporine A; n.s., not significant; ***p < 0.001,**p < 0.01, *p < 0.05, Kruskal-Wallis Test with Dunn’s correction, scale bar equals 250 μm).

We also assessed the morphology of the colon mucosa by histological analyses. Colon tissue of DSS-treated mice showed abundant mucosal erosions with a disrupted epithelial layer and abundant infiltration of leukocytes (Figures 4A,B; Supplementary Figure S2). Treatment with either voclosporin or cyclosporine A significantly reduced mucosal erosions and leukocyte infiltration. The constitution of the intestinal epithelium in DSS-treated mice was further analyzed by immunofluorescence. Analysis of E-Cadherin demonstrated a marked disruption of the epithelial layer in DSS-treated mice. Calcineurin inhibition by either voclosporin or cyclosporine A protected the intestinal epithelium as less erosions were present in these mice (Figures 4C,D; Supplementary Figure S3). The mucus and goblet cells were analyzed by UEA-1. This revealed the marked loss of goblet cells in the course of DSS-induced colitis. Protection from epithelial damage was evident in both voclosporin and cyclosporine A-treated mice but was even more pronounced in voclosporin-treated mice which featured hardly any epithelial damage or goblet cell loss (Figures 4E,F; Supplementary Figure S4). In association with the damaged epithelium, marked neutrophil infiltration occurred in the course of DSS-induced colitis in solvent control-treated mice as evidenced by neutrophil elastase immunofluorescence (Figures 4G,H; Supplementary Figure S5). Calcineurin inhibition by either voclosporin or cyclosporine A strongly diminished neutrophil influx in DSS-treated mice. Taken together, both voclosporin and cyclosporine A are capable of strongly alleviating mucosal damage in the course of DSS-induced colitis. Calcineurin inhibition protects from epithelial damage, goblet cell loss and diminishes neutrophil influx. Based on its improved drug safety profile, voclosporin could be clinically developed for the treatment of severe ulcerative colitis.

## Discussion

Acute steroid-refractory colitis (ASUC) represents a critical clinical condition which requires prompt response to medical rescue treatment to avoid colectomy or even mortality. In a recent United Kingdom-wide audit, mortality occurred in 0.7%–1.2% of hospital admissions due to severe ulcerative colitis. 60% of patients responded to steroid therapy but approximately 50% of these patients received additional second-line treatment and up to 20% had to undergo surgery (11). Thus, there is an unmet need to increase therapeutic options in this situation. Cyclosporine A, tacrolimus and the anti-TNF antibody infliximab have constituted the mainstay of therapy in the treatment of acute severe ulcerative colitis once steroids have failed. Additionally, JAK inhibitors are increasingly approved for the use in moderate to severe ulcerative colitis (12). While some physicians may favor infliximab as compared to cyclosporine A for reasons of drug safety, there is a high rate of non-response to infliximab in acute severe ulcerative colitis (13). Clinical features that predict failure of anti-TNF therapy include elevated CRP, low serum albumin and low serum infliximab levels shortly after therapy induction (14). One explanation for the failure of anti-TNF in this clinical setting is loss of the compound via the fecal route (15). In this setting small molecules may be advantageous as compared to biologicals and may serve as a bridge to biological therapy (16). Given the recent success of voclosporin in the treatment of lupus nephritis (17) and its potentially improved safety profile (9), we hypothesized a potential role for voclosporin in the treatment of acute steroid-refractory ulcerative colitis (18).

As a model system we first assessed the effects of voclosporin and cyclosporine A on isolated human CD4^+^ T lymphocytes from healthy subjects *in vitro*. As expected, both voclosporin and cyclosporine A effectively reduced the expression of multiple cytokines by T cells including IL-2, IL-10, IL-17A, IL17F and TNF-α on both RNA and protein level after both anti-CD3/CD28 and PMA/ionomycin stimulation. In contrast, *CASP8*, *ITK* and *MALT1* were shown to be upregulated. The activation of Interleukin-2-inducible T cell kinase (ITK) was recently shown to be inhibited by cyclosporine A and critically affects the efficacy of calcineurin inhibition in oxazolone-induced colitis (19). Flow cytometric analyses showed that both calcineurin inhibitors abrogated the response to polyclonal TCR stimulation as assessed by a reduction in lymphocytes expressing the activation markers CD25, CD44 or CD69. Overall, these analyses showed similar effects of voclosporin and cyclosporine A in blocking T cell activation and cytokine production *in vitro*. No specific functional difference between the two compounds was detected in these assays. Previously, voclosporin had been identified as being even more potent *in vitro* than cyclosporine A (20). Our experiments do not contradict these findings, as the least effective dose was not titrated in our approaches. In this project, we focused our *in vitro* analyses on T lymphocytes as a model cell type to assess the intracellular effects of voclosporin and cyclosporine A. However, the effects of calcineurin inhibition are not limited to T cells. In addition to lymphocytes, calcineurin and NFAT signaling has been reported in both tissue resident cells, e.g., intestinal epithelial cells (21–23), as well as in cells of the myeloid lineage (24, 25). So far, it has not been fully elucidated whether the strong therapeutic effect of calcineurin inhibitors is linked to effects in a certain cell type. Likely, there are tissue-dependent and model-dependent effects, which determine the respective contribution of each cell type (19).

Next, we assessed the therapeutic efficacy of both cyclosporine A and voclosporin in a well-established experimental model of severe colitis in mice induced by dextran sodium sulfate. We chose the dextran sodium sulfate-model of acute colitis, which shares many clinical features with acute severe ulcerative colitis, especially in regard to mucosal erosions, rectal bleeding and diarrhea development. Various studies have previously described protective effects of cyclosporine A in DSS-induced colitis (22, 23, 26). We observed that both voclosporin and cyclosporine A strongly abrogated severe colitis in this model. Overall disease activity, weight loss and mucosal damage as assessed by histology were ameliorated by both drugs to a similar extent. Additionally, calcineurin inhibition reduced mucosal calprotectin levels as assessed by qPCR. Immunofluorescence revealed frequent disruptions of the epithelial layer in DSS-treated mice. Inhibition of calcineurin protected from epithelial damage and goblet cell loss and markedly reduced leukocyte infiltrations which stabilize mucosal erosions (27). This is in line with observations that point to a direct role of calcineurin in intestinal epithelial cell death (22, 23), which is critical during DSS-induced colitis (28, 29). Our histological analyses point to a more consistent abrogation of mucosal damage, epithelial dysfunction and goblet cell loss in voclosporin-treated mice. This may be the result of the proposedly increased potency. However, in this pilot study the dosage was not systematically titrated. Thus, these experiments do not provide enough evidence to favor one calcineurin inhibitor over the other. The assessment of drug toxicity is species-specific (30). Previous studies have studied the safety profile of voclosporin in the course of the development for its use in lupus nephritis (8). The optimal effective dose for its use in ulcerative colitis should ideally be systematically assessed before applying voclosporin in the context of acute severe ulcerative colitis.

Voclosporin proved as effective as cyclosporine A in abrogating T cell activation and cytokine production *in vitro* and strongly attenuated severity of experimental colitis *in vivo*. While no specific pharmacodynamic differences were noted, advantages in pharmacokinetics have been noted with regard to reduction in toxic metabolites, no need for therapeutic drug monitoring and thus potentially an increased ease of use (Table 1). Both cyclosporine A and voclosporin are largely metabolized by CYP3A4 (9), so attention to potential drug interactions needs to be paid. As with cyclosporine A, the U.S. Food and Drug Administration notes a potentially increased risk of developing malignancies and serious infections associated with voclosporin or other immunosuppressants. Dose adjustment in impaired renal function is necessary and blood pressure should be checked regularly under treatment (31) Pharmacokinetic advances, such as an increased bioavailability of cyclosporine A in the neoral/optoral formulations had a relevant impact on patient care in the past (32). Potentially limiting cost-effectiveness should be overcome in the upcoming years (33). From a medicolegal point of view, acute severe ulcerative colitis is not an approved indication for cyclosporine A (34). So, despite decades of experience with cyclosporine A and favorable recommendations in treatment guidelines, both voclosporin and cyclosporine A use in ulcerative colitis would be considered off-label. Future studies are warranted to find whether therapeutic efficacy of voclosporin in experimental colitis translates to the treatment of acute severe ulcerative colitis. Furthermore, additional therapeutic options arise as more drugs are being approved for this disease and evaluated in severe cases. In recent years, Janus kinase (JAK) inhibitors, e.g., tofacitinib, filgotinib and upadacitinib (35–37), have been approved as treatment alternatives in moderate to severe ulcerative colitis. Currently, there is no head-to-head comparison of the efficacy of JAK inhibitors to other treatment options in this indication (38). Recent studies suggest effectiveness of tofacitinib in biologic-experienced hospitalized patients with acute severe ulcerative colitis, but more studies are needed (12). The TOCASU trial is listed as NCT05112263 and aims to compare tofacitinib to cyclosporine A in acute severe ulcerative colitis.

**Table 1:** Comparison of various properties of cyclosporine A and voclosporin.

Feature	Cyclosporine A	Voclosporin
Mode of action	Calcineurin inhibition	Calcineurin inhibition
Clinical experience	+++	−
FDA/EMA approval	+	+
Approval for ulcerative colitis (26)	−	−
Cost	Low	High
Toxic metabolites (9)	Yes	Reduced
Drug metabolism (9)	CYP 3A4	CYP 3A4
Potency (9)	+	++
Therapeutic drug monitoring (10)	Needed	In question

The calcineurin inhibitors cyclosporine A and voclosporin share structural similarities and mode of action, but feature significant differences with regard to drug metabolism, cost effectiveness, toxicity, potency or label of use. This table highlights similarities and differences of both compounds.

Taken together, voclosporin is as effective as cyclosporine A in abrogating DSS-induced colitis in mice. Voclosporin might be further developed as an alternative agent for the treatment of severe steroid-refractory ulcerative colitis with advantages in clinical management and ease of use with reduced toxicity and improved safety profile compared to cyclosporine A.

## Data availability statement

The raw data supporting the conclusions of this article will be made available by the authors, without undue reservation.

## Ethics statement

The studies involving human participants were reviewed and approved by Ethikkommission der Universitätsklinik Erlangen. The patients/participants provided their written informed consent to participate in this study. The animal study was reviewed and approved by Regierung von Unterfranken.

## Author contributions

AL, DR, and KK performed the experiments. ML designed the study. AL and ML drafted the manuscript. All authors contributed to the article and approved the submitted version.

## Funding

This work has been supported by the Deutsche Forschungsgemeinschaft (DFG, German Research Foundation—Project-IDs 375876048—TRR 241 (A08, B04, B08, Z03); CRC1181-C02 (261193037), NE1927/2-2, and FOR2438-P09 (280163318) to CN, ZU377/4-1 to SZ) and the Interdisciplinary Center for Clinical Research (IZKF) of the Friedrich Alexander University Erlangen-Nürnberg (J85) to KK.

## Conflict of interest

The authors declare that the research was conducted in the absence of any commercial or financial relationships that could be construed as a potential conflict of interest.

## Publisher’s note

All claims expressed in this article are solely those of the authors and do not necessarily represent those of their affiliated organizations, or those of the publisher, the editors and the reviewers. Any product that may be evaluated in this article, or claim that may be made by its manufacturer, is not guaranteed or endorsed by the publisher.
