# Supplementary material for: Protective effect of the novel calcineurin inhibitor voclosporin in experimental colitis
Source: Front Med (Lausanne). 2023 Jun 9;10:1177450. doi: 10.3389/fmed.2023.1177450 (PMC10289195; doi:10.3389/fmed.2023.1177450)
Supplement: Supplementary file 2 [file Data_Sheet_1.pdf]

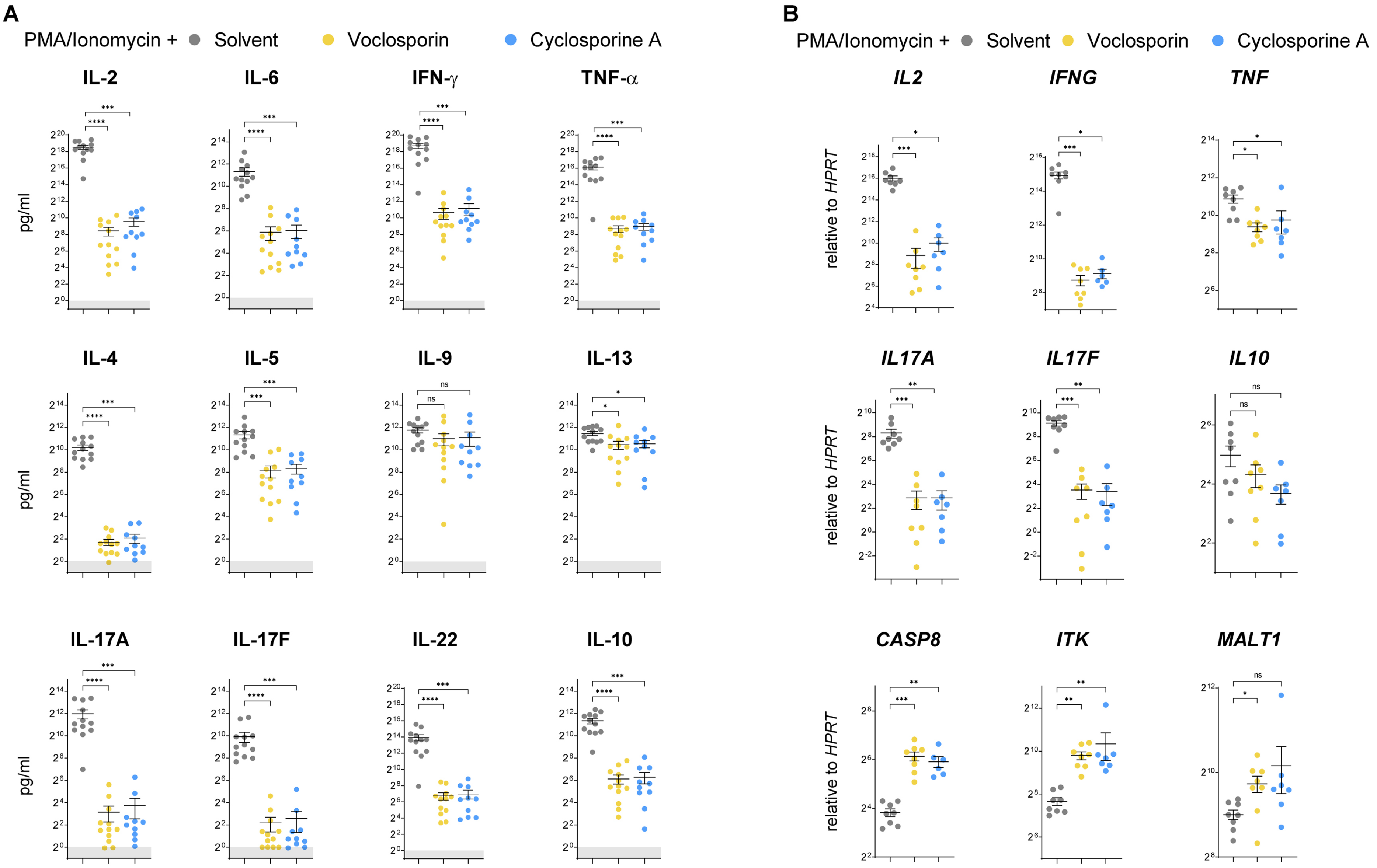
A
PMA/Ionomycin +
Solvent
Voclosporin
Cyclosporine A
IL-2
IL-6
IFN-γ
TNF-α
IL-4
IL-5
IL-9
IL-13
IL-17A
IL-17F
IL-22
IL-10
pg/ml
B
PMA/Ionomycin +
Solvent
Voclosporin
Cyclosporine A
IL2
IFNG
TNF
IL17A
IL17F
IL10
CASP8
ITK
MALT1
relative to HPRT

Figure S1: Calcineurin inhibition by either cyclosporine A or voclosporin abrogates the production of multiple T cell-derived cytokines

(A, B) Human peripheral blood T lymphocytes were isolated from normal healthy donors and stimulated with PMA and ionomycin for 48 hours in the presence or absence (solvent control, grey) of the calcineurin inhibitors voclosporin (10 µg/ml, yellow) or cyclosporine A (10 µg/ml, blue). Consecutively, culture supernatants were analyzed for cytokine secretion and T cell RNA was isolated and subjected to qPCR analyses. (A) In stimulated T cells, ample amounts of IL-2, IL-6, IFN-γ, TNF-α, IL-4, IL-5, IL-9, IL-13, IL-17A, IL-17F, IL-22 and IL-10 are produced which is abrogated in the presence of either voclosporin or cyclosporine A (N=10 donors, pooled analysis of at least three independent experiments). (B) qPCR analysis of *IL2*, *IFNG*, *TNF*, *IL17A*, *IL17F* and *IL10* revealed that not only secretion but also induction of transcription of these cytokines is abrogated in the presence of either voclosporin or cyclosporine A. In contrast, expression of *CASP8*, *ITK* and *MALT1* is elevated in the presence of either calcineurin inhibitor. (N = 8 donors, pooled analysis of at least three independent experiments) (**** $p < 0.001$, *** $p < 0.001$, ** $p < 0.01$, * $p < 0.05$, Kruskal-Wallis-Test with Dunn's correction).

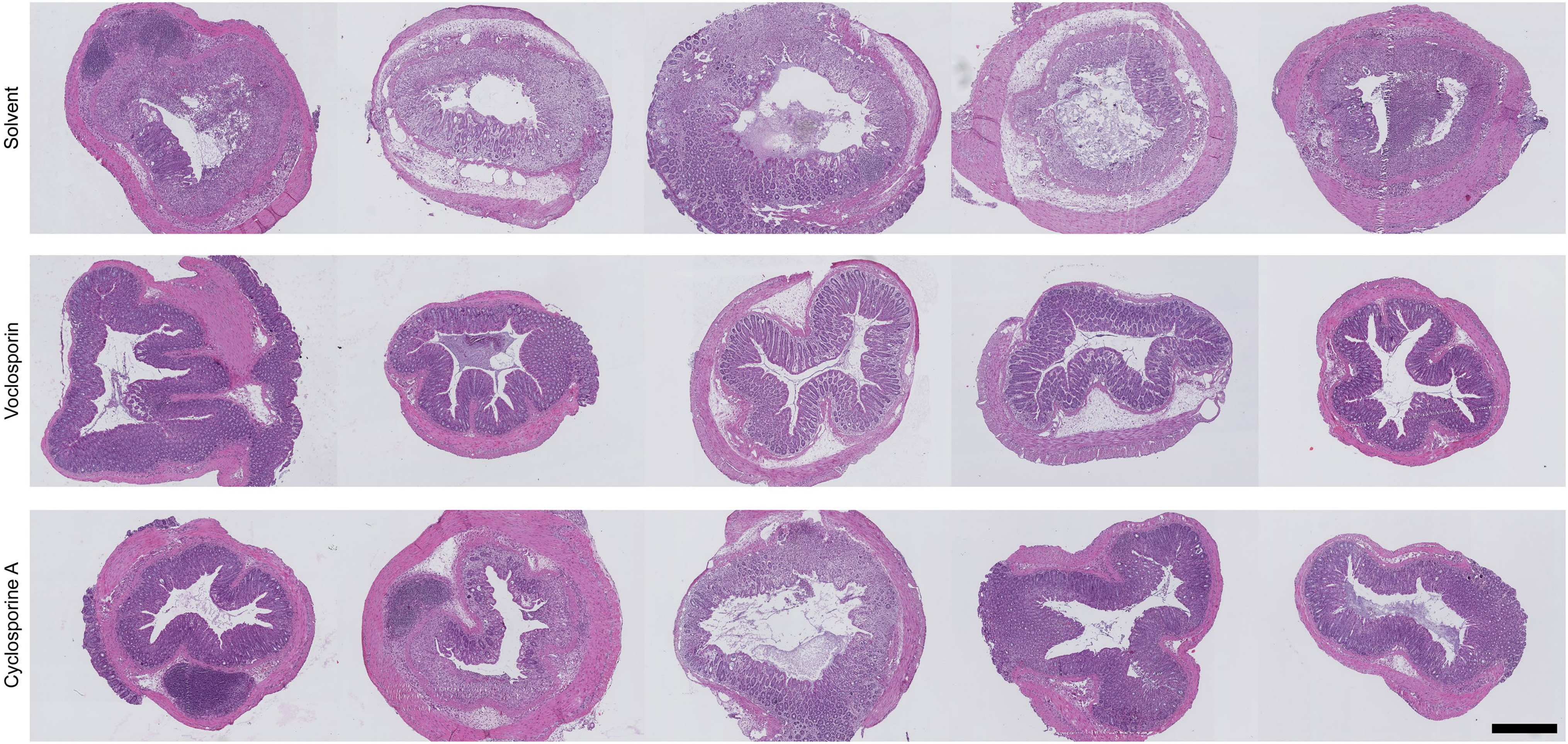
Solvent
Voclosporin
Cyclosporine A

Figure S2: Calcineurin inhibition by either cyclosporine A or voclosporin markedly alleviates mucosal damage in DSS-induced colitis

An overview of the histological analyses by hematoxylin & eosin of colon tissue sections of mice sacrificed on day 9 is depicted (N = 5 mice per group). Scale bar represents 500 µm.

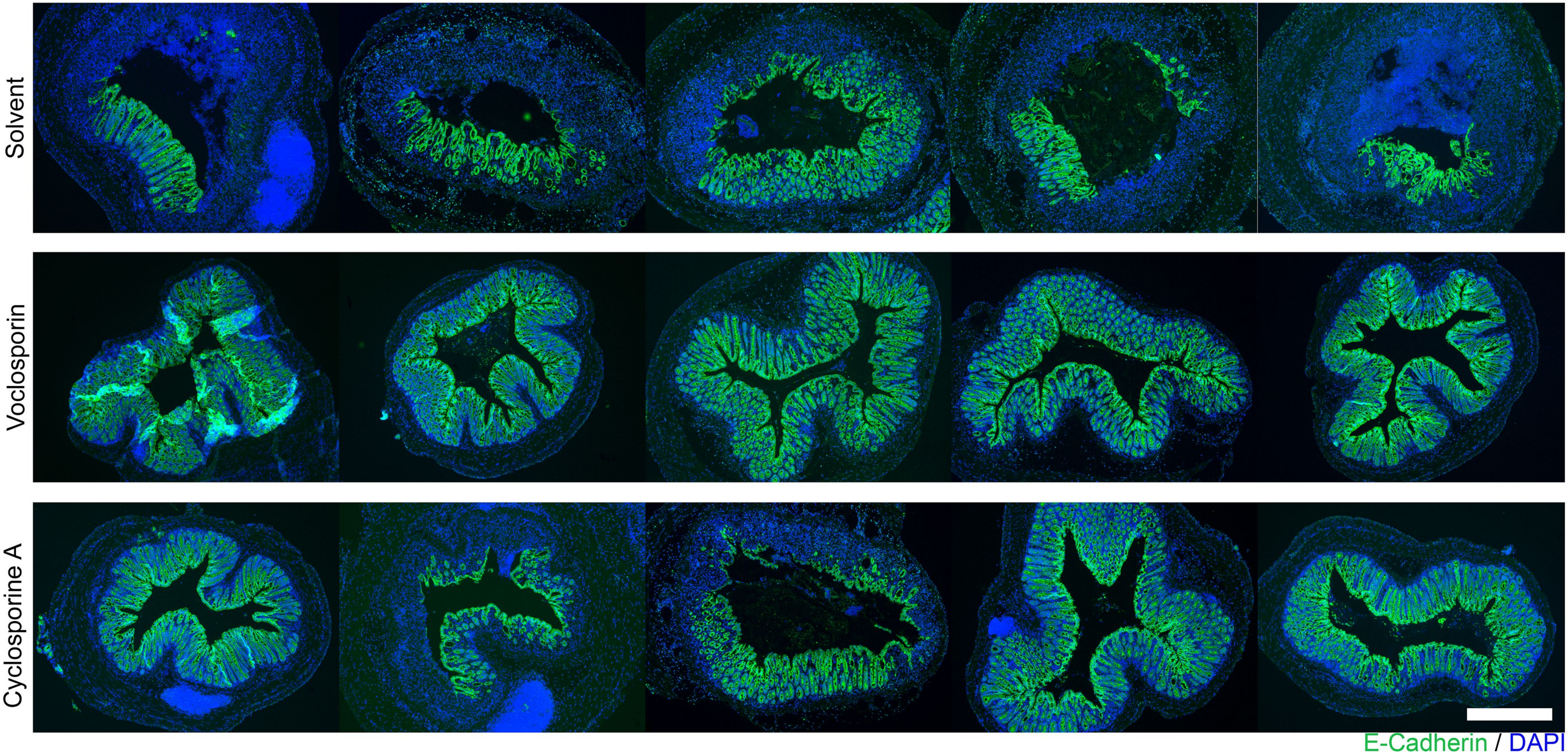
Solvent
Voclosporin
Cyclosporine A
E-Cadherin / DAPI

Figure S3: Calcineurin inhibition by either cyclosporine A or voclosporin protects the epithelial layer in DSS-induced colitis

An overview of the histological analyses of the intestinal epithelial layer by E-Cadherin immunofluorescence of colon tissue sections of mice sacrificed on day 9 is depicted (N = 5 mice per group). Scale bar represents 500 µm.

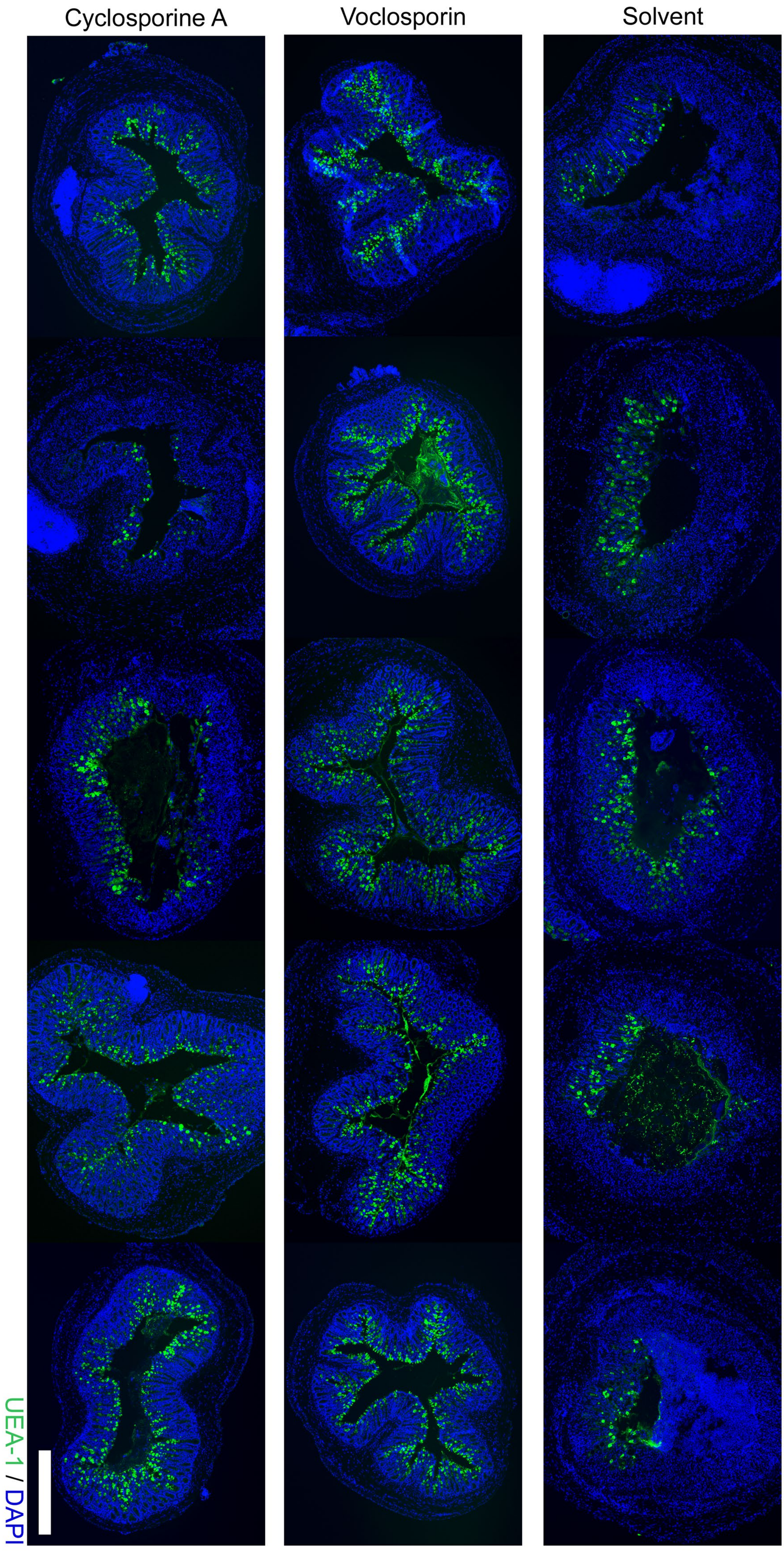
Cyclosporine A
Voclosporin
Solvent
UEA-1 / DAPI

Figure S4: Calcineurin inhibition by either cyclosporine A or voclosporin attenuates goblet cell loss in DSS-induced colitis

An overview of the histological analyses of goblet cells and the mucus layer by UEA-1 immunofluorescence of colon tissue sections of mice sacrificed on day 9 is depicted (N = 5 mice per group). Scale bar represents 500 µm.

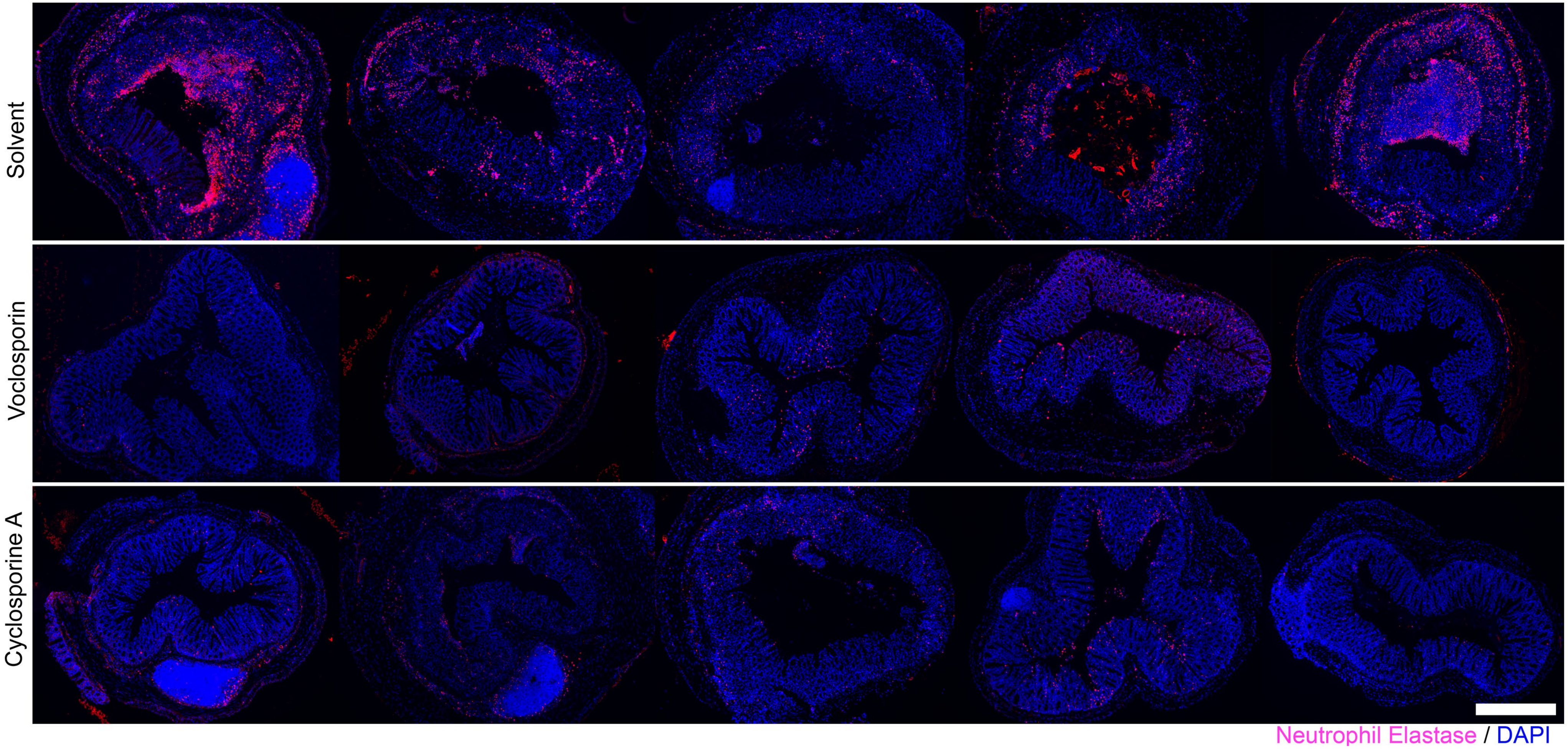
Solvent
Voclosporin
Cyclosporine A
Neutrophil Elastase / DAPI

Figure S5: Calcineurin inhibition by either cyclosporine A or voclosporin attenuates neutrophil infiltration in DSS-induced colitis

An overview of the histological analyses of neutrophil infiltration by neutrophil elastase immunofluorescence of colon tissue sections of mice sacrificed on day 9 is depicted (N = 5 mice per group). Scale bar represents 500 µm.

Movie 1: Calcineurin inhibition by either cyclosporine A or voclosporin attenuates DSS-induced colitis

Mini-colonoscopy of a healthy mouse as compared to DSS-treated mice treated with solvent control, voclosporin or cyclosporine A is presented. Please appreciate perianal blood in solvent control-treated mice, as well as diarrhea and mucosal erosions, which are almost absent in mice treated with either calcineurin inhibitor.
